# Evaluation of Performance of Existing RO Drinking Water Stations in the North Central Province, Sri Lanka

**DOI:** 10.3390/membranes11060383

**Published:** 2021-05-24

**Authors:** Suresh Indika, Yuansong Wei, Dazhou Hu, Jegetheeswaran Ketharani, Tharindu Ritigala, Titus Cooray, M. A. C. K. Hansima, Madhubashini Makehelwala, K. B. S. N. Jinadasa, Sujithra K. Weragoda, Rohan Weerasooriya

**Affiliations:** 1 State Key Joint Laboratory of Environmental Simulation and Pollution Control, Research Center for Eco-Environmental Sciences, Chinese Academy of Sciences, Beijing 100085, China; indika_st@rcees.ac.cn (S.I.); dzhu_st@rcees.ac.cn (D.H.); tharindu_st@rcees.ac.cn (T.R.); 2 Laboratory of Water Pollution Control Technology, Research Center for Eco-Environmental Sciences, Chinese Academy of Sciences, Beijing 100085, China; 3 University of Chinese Academy of Sciences, Beijing 100049, China; 4 National Institute of Fundamental Studies, Hanthana Road, Kandy 20000, Sri Lanka; rohan.we@nifs.ac.lk; 5 Department of Civil Engineering, University of Peradeniya, Peradeniya 20400, Sri Lanka; ketha94@eng.pdn.ac.lk (J.K.); shamj@pdn.ac.lk (K.B.S.N.J.); 6 Department of Applied Earth Sciences, Uva Wellassa University, Badulla 90000, Sri Lanka; titus@uwu.ac.lk; 7 Post Graduate Institute of Science (PGIS), University of Peradeniya, Peradeniya 20400, Sri Lanka; 127charithahansima@gmail.com; 8 China-Sri Lanka Joint Research and Demonstration Center for Water Technology, Ministry of Water Supply, Meewathura, Peradeniya 20400, Sri Lanka; madhu10w@yahoo.com; 9 National Water Supply and Drainage Board, Katugastota 20800, Sri Lanka; skwera7@gmail.com

**Keywords:** reverse osmosis, performance, selectivity, salt rejection, permeate water recovery

## Abstract

Reverse osmosis (RO) drinking water stations have been introduced to provide safe drinking water for areas with prevailing chronic kidney disease with unknown (CKDu) etiology in the dry zone of Sri Lanka. In this investigation, RO drinking water stations established by community-based organizations (CBO) in the North Central Province (NCP) were examined. Water samples were collected from source, permeate, and concentrate in each station to determine water quality and performance. Furthermore, the operators of the systems were interviewed to evaluate operational and maintenance practices to identify major issues related to the RO systems. Results show that the majority (>93%) of RO systems had higher salt rejection rates (>92%), while water recovery varied from 19.4% to 64%. The removal efficiencies of hardness and alkalinity were averaged at 95.8% and 86.6%, respectively. Most dominant ions such as Ca^2+^, Mg^2+^, K^+^, Na^+^, Ba^2+^, Sr^2+^ Cl^−^, F^−^, and SO_4_^2−^ showed higher rejections at averaged values of 93.5%, 97.4%, 86.6%, 90.8%, 95.4%, 96.3%, 95.7%, 96.6%, and 99.0%, respectively. Low recovery rates, lower fluoride levels in product water, and membrane fouling were the main challenges. Lack of knowledge and training were the major issues that could shorten the lifespan of RO systems.

## 1. Introduction

Safe and clean drinking water demand has been exponentially increasing around the globe due to the water quality degradation by environmental pollution as well as depletion of natural water bodies with the climate change. Even though Sri Lanka has not been an industrialized country, anthropogenic activities such as agricultural practices and waste disposal strategies have significantly influenced the quality of freshwater bodies in certain areas other than natural and environmental factors. In particular, rural communities in the dry zone of Sri Lanka, which cover two-thirds of the island, have suffered from severe health issues regarding the consumption of groundwater [1,2,3,4]. The prevalence of CKDu has been a life-threatening crisis for the farming community in the dry zone [5,6]. Until now, none studies have been conducted giving the exact cause for this disease. However, it has been emphasized that the drinking water quality issue might be the major cause of the origin of kidney disease [7,8]. Generally, the major drinking water source of the farming community in the dry zone had been raw groundwater for several decades. The conventional water treatment technologies used in Sri Lanka do not have the capability to remove excess dissolved minerals and possible nephrotoxins in the high hard groundwater in the dry zone. Therefore, advanced drinking water purification technologies such as RO membrane technology have been introduced and widely spread throughout the dry zone in the last decade to improve the drinking water quality [9,10].

Generally, more than 2000 RO drinking water stations have been established by different organizations [9,11], including government, non-government (NGOs), and some private companies throughout the north-central province (NCP). The CBOs, the Sri Lankan navy, the civil security department, and private companies mainly operate drinking water stations in the dry zone. The averaged treatment capacity of the systems ranges from 7.5 to 15 m^3^ per day. Moreover, different RO configurations, pre-treatment technologies, and post-treatment processes are available in this study area. Similarly, different operational and maintenance practices are followed by the operators in these RO stations. Previous studies have reported that many RO stations were not following proper or standard guidelines for operational practices. Thus, it has become a major problem that causes lower product water quality and reduced lifespan of the RO systems [9].

High removal rates of dissolved minerals and contaminants are the major advantage of the RO technology for drinking water production [12]. The production capability of soft drinking water from unpalatable high hard groundwater common in NCP has been significantly valuable. The existing RO stations in NCP have reported sufficiently high removal or rejection efficiencies (91–98%) for major constituents such as TDS, hardness, alkalinity, silica, and major ions (Ca^2+^, Mg^2+^, Na^+^, Cl^−^, and SO_4_^2−^) in the groundwater [10]. However, lack of essential minerals in the product RO water has been identified as a major drawback, leading to health issues related to nutrient deficiencies [13,14]. Most RO stations in NCP have been reported as having lower levels of essential minerals in the product water such as Na, K, Ca, Mg, and fluoride, which cause water palatability issues and mineral deficiency health issues such as dental decay [15,16,17]. Recently, other than inorganic constituents reported, the health effect from the dissolved organic fraction (DOC) in the groundwater has become a novel hypothesis for the CKDu issue in the dry zone [18]. Moreover, certain previous studies emphasized the unacceptable levels of DOC in the NCP region [8,18,19], and an average of 55% of organic matter removal was observed in the RO systems in NCP [10]. Community feedback showed that the number of newly reported CKDu patients had been drastically reduced as an indication of improvement of community well-being by this drinking water treatment method. Therefore, to overcome the drawbacks such as higher removal of the essential minerals, previous studies have recommended advanced membrane technologies such as nano filtration (NF) and electro-dialysis reversal (EDR) water treatment strategies [15,20].

RO membrane technology for drinking water purification has been very popular within the rural communities in the dry zone of Sri Lanka, especially after discovering CKDu disease. Although these RO systems in these regions are the major drinking water source that have already become widespread within last decade, thus far, no comprehensive study has been conducted to evaluate their performance, operational issues, and product water quality, except in a few general investigations based on a few RO stations [9,10]. Hence, the main objective of this study was to comprehensively evaluate the performance of CBO-established RO stations on the basis of their salt rejection capability and permeate water recovery. Identification of major operational and maintenance practices was the second objective. Furthermore, identification of product water quality issues, as well as estimation of production cost for RO drinking water in NCP, were carried out as final objectives. Thus, this study showed proper guidelines for future investigations regarding implementing and maintaining novel membrane technologies such as RO, NF, and EDR in Sri Lanka to supply clean and safe drinking water to the community.

## 2. Materials and Methods

### 2.1. Study Area

**Figure 1:**
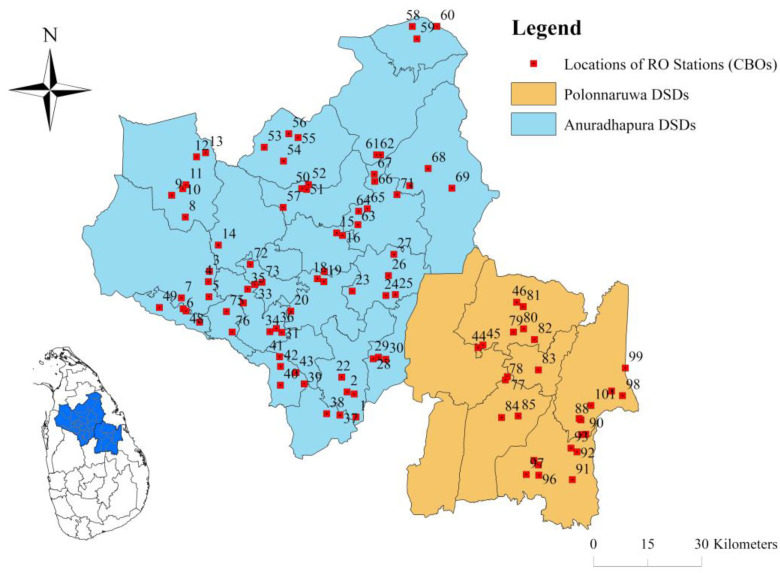
Locations of CBO-operated RO stations in North Central Province, Sri Lanka.

Anuradhapura and Polonnaruwa, which belong to the NCP in Sri Lanka, are the most affected regions by the CKDu health crisis. The quality of groundwater bodies in these regions is suspected to be the cause of this issue. Hence, NCP was selected as the study area, including all divisional secretariats (DSS) in Anuradhapura (22) and Polonnaruwa in this investigation. CBO-operated RO stations are the most common RO drinking water stations in the rural community of the NCP. Therefore, CBO-operated medium- and small-scale RO drinking water stations installed in NCP were investigated, and 101 units of stations in NCP were selected by screening with the help of information given by NWSDB to represent all the CKDu prevailing regions in NCP. Out of them, 73 were from Anuradhapura, and the rest were from Polonnaruwa. Figure 1 shows locations of the investigated RO stations in NCP.

### 2.2. Sampling and Data Collection

A total of 101 units of RO systems (100 CBOs) were examined, and three major types of water samples (source, permeate, concentrate) were collected for water quality analysis to evaluate performance of each RO system. Collection of operational and maintenance practice information of each station was conducted by a questionnaire filled out by the operator who was responsible for the operation of the RO station. The operation conditions and system specification data were collected by direct observation. The questionnaire was focused on the RO system’s basic information and treatment process, operational and maintenance practice, performance information, cost and income data, concentrate disposal, consumer feedback and comments, challenges, and recommendations. The consumer feedback survey was generally focused on their attitudes and opinions on RO drinking water quality, giving attention on whether it would be beneficial for their health to mitigate the CKDu issue in that area, whether it is consumable, would they have any problem with the taste or smell of RO drinking water, and what are the misconceptions about the RO drinking water. GPS locations and photographic information of all the RO systems were captured.

### 2.3. Water Quality Analysis

All the RO water samples were analyzed in the Environmental Laboratory, Faculty of Engineering, University of Peradeniya, Sri Lanka; National Water Supply and Drainage Board (NWSDB) Katugastota, Kandy; and NIFS laboratory Kandy for physical water quality parameters (pH, EC, TDS) and chemical parameters (hardness, alkalinity, metal cations, and anions). pH and EC of the water samples were analyzed by the Thermo Scientific Orion Star A325 Multiparameter meter. All the dominant and trace metals (Ca^2+^, Mg^2+^, Na^+^, K^+^, Li^+^, Sr^2+^, Ba^2+^, Mn^2+^, Fe^2+^, Cd^2+^, As^3+^, Cu^2+^, Zn^2+^, Cr^3+^, Hg^2+^, and Si) concentrations were measured by an inductively coupled plasma optical emission spectrophotometer (iCAP 7000 Series ICP-OES, Thermo Fisher Scientific, Waltham, MA, USA) in NIFS. The common anions (Cl^−^, F^−^, Br^−^, SO_4_^2−^, NO_3_^−^, and PO_4_^3−^) were analyzed by an ion-chromatography instrument (Eco IC, Metrohm, Sweden) in the NWSDB. The total alkalinity was tested by the titrimetric method [21], and the total hardness was determined by the Ca^2+^ and Mg^2+^ concentrations in the water samples using the following equation:

Total hardness (mg/L CaCO_3_) = 2.497 × [Ca](mg/L) + 4.116 × [Mg](mg/L) (1)

### 2.4. Data Analysis

Mineral (salt, hardness, alkalinity, cations, anions) rejections, permeate recovery, and ion permeability of RO membranes were used as the major performance criteria of the RO systems. Operational data collected by the questionnaires, manufacturer datasheets, and direct observation of the site were used to compare the actual operating conditions and standard conditions that are given by the manufacturers to identify the major issues with the operational and maintenance practices. Information related to the economy of the CBOs such as income, energy costs, replacement and chemical costs, and salaries were used to estimate the rough production cost for the RO drinking water. Origin Pro 2018 (64-bit) SR1 n9.5.1.195 (OriginLab Corporation, Northampton, MA, USA) and IBM SPSS Statistics for Windows, Version 26.0 (Armonk, NY, USA: IBM Corporation) software were used to analyze as a whole the water quality and other data in order to interpret it by the charts and diagrams. ArcGIS 10.3 for Desktop, Version 10.3.0.4322 (Esri Inc., Redlands, CA, USA) software was used to create maps related to this investigation. Equations for calculations of rejection and recovery are given below.

% Recovery = (permeate flow/feed flow) × 100%(2)

% Rejection = [(C_f_ − C_p_)/C_f_] × 100% (3)

where

C_f_: influent concentration of a specific component, mg/L;

C_p_: permeate concentration of a specific component, mg/L.

## 3. Results and Discussion

### 3.1. Comparison of RO Treatment Process

There were different models of RO systems manufactured and distributed by different companies operating under different CBOs in the study area (Table S1). The basic filtration process was versatile in process configuration, pre-treatment methods, post-treatment methods, capacity, membrane type/manufacturer, reject-water handling, etc. Figure S1 shows the process flow diagram for a typical RO system in NCP.

#### 3.1.1. Pre-Treatment Process

The main pre-treatment units were used with sand/multimedia filtration, activated carbon (AC) filtration, water softening, and sediment filtration (MF or UF cartridges). The pre-treatment process plays a crucial role in order to avoid fouling and scaling on more expensive RO membranes due to silt or sediment [22]. The water softening unit was not available in the majority of RO systems. Only 36% of RO stations (out of 101 RO systems) contained a water softening unit. Sediment filters used in CBO-operated RO stations use two types of MF or UF cartridge, normally with 5-micron pore sizes. They are polypropylene (PP) poly spun melt blown and PP string-wound sediment filter cartridges.

A considerable number of RO systems (>44%) use antiscalants to minimize RO membrane fouling and scaling. Two major antiscalant types with two different manufacturers were observed in the CBO-operated RO stations in NCP. The performance of these two regents was not evaluated. However, the dosage of antiscalant varied site by site. The antiscalant solution is usually injected into the pretreated (after sand, AC, and softener) water before the sediment cartridge filter (MF), which has been assembled before the high-pressure pump. The majority of operators use a solution with 50 mL of antiscalants dissolved in 100 L water for one week of production (8400 L).

Even though the antiscalant process is very common in RO stations in the NCP, it was observed that the majority of the operators do not have a clear idea about the purpose of the antiscalant. Due to this reason, some of the operators have stopped adding antiscalant because of the misunderstanding about the antiscalant as a chemical component that can be toxic for long-term human consumption. A significant number of consumers have a negative opinion about the chemicals added to drinking water in that they would lead to serious health effects. Thus, some of the RO stations have stopped adding minerals to RO filtered water in their re-mineralization process as well as in their antiscalant dosing process.

#### 3.1.2. RO Filtration

All the RO stations in the study area use spiral wound membrane elements. The majority of them have the size of 4′′ × 40′′ RO elements. The number of RO skids and the configurations vary widely and depend on the capacity of the RO system. Figure S2 illustrates the most common configurations of RO systems in this region.

According to the number of stages, categorization of the RO systems can be done as shown in Table S2. We identified that the most common types of RO systems are single-stage with single RO element, two-stage serial arrangement with two RO elements, two-stage Christmas tree arrangement, and three-stage serial-type with three RO elements.

Apart from the configuration, different types or models of the RO membranes used in CBOs of the NCP showed slightly different performances, even though they are made of the same polyamide material with TFC structure (Table S3). The most common RO membrane types used in RO systems in the NCP are manufactured by Hydranautics, VONTRON, and DOW filmtec. Usually, the pure aqua RO system uses Hydranautics (ESPA2-LD-4040) membranes inside their pressure vessels.

Usually, RO membranes used in the NCP are specified for different pressure ranges for different membrane models. On the basis of the tested operating pressure decided by the manufacturer to achieve 15% of permeate recovery per membrane element, we can categorize them into three main types of RO membranes: low pressure (LP), ultra-low pressure (ULP), and extremely low pressure (XLP) RO membranes.

#### 3.1.3. Post-Treatment Process

Generally, ultraviolet (UV) radiation and remineralization units are used as the post-treatment processes for product permeate water. UV radiation has been widely used for the disinfection process for RO drinking water production while replacing the conventional chlorination process that can lead to the formation of carcinogenic disinfection by-products such as tri-halo-methane. Around 39.6% of RO stations contained UV disinfection units and around 7.9% of ROs used a re-mineralization unit. The RO systems that produced permeate with very low TDS or EC level (TDS < 10 ppm) use the remineralization unit as a mineral adjustment method. Two major remineralization processes that are common in NCP are injecting pre-dissolved mineral solution into the permeate by an automatic dosing system and filtering RO permeate through the calcite bed/contactor. Two RO stations were observed as having calcite bed mineral addition.

#### 3.1.4. Reject Water Handling

Production of wastewater (concentrate/reject water) from these RO stations was quite significant due to their lower recovery rates (<50%). The majority of the CBO-established RO stations did not have a proper wastewater disposal method. A total of 80% of RO stations simply reject their concentrated wastewater into open soil (Figure S3). However, only 10% of RO systems were found to consist of a reject water treatment unit such as multimedia filter. A few RO stations use their wastewater for gardening or cement construction purposes. However, some of the RO operators complained that RO wastewater is killing their cultivations. Therefore, they have stopped using wastewater for gardening purposes. Some villagers do not use the RO reject water as irrigation water for crops due to believing a myth that vegetables and fruits that are produced or cultivated by using wastewater from ROs become toxic and not suitable for human consumption. Hence, the feasibility of this RO reject water for irrigation purposes should be checked in future studies by analyzing different irrigation water quality measures.

### 3.2. Operation and Maintenance

CBO-operated RO stations do not have a standard operating guideline due to each RO system being donated/purchased and maintained by different organizations [9,10]. However, some organizations have provided detailed guidelines to some of the RO stations. Yet, it cannot be guaranteed that most of the RO operators are following the proper guideline for the operations of the RO systems. The NWSDB provides basic operational training for all the RO operators. Most of the CBO-operated RO stations have been under consultation and maintenance by NGOs other than NWSDB.

According to the information gathered during this study, the majority of the RO systems operated under CBOs have been installed after 2013. Thus, most of them are no more than eight years old (Figure S4). Hence, this technology is still new to the Sri Lankan water sector. Significantly lower daily operational time durations were observed from the RO stations in the NCP due to the lower demand for the drinking water from the CBO-operated RO systems. The average time of 4.4 h per day was observed, ranging from 1 to 24 h/day. The majority of the RO operators are farmers and do not have proper technical knowledge about operating and maintenance of the RO systems. Generally, RO operators do not involve extensive repairs in the RO system, such as replacing RO membrane modules, sand/activated carbon/resins in pre-filters, and pumps. Those tasks are usually performed by maintenance agents in the NWSDB or relevant authorities.

Operation and maintenance of these pre-treatment units are crucial for drinking water quality and the RO membrane life span, which directly influence the economy of the station. Around 72% of CBO-operated RO stations use daily backwashing routines (mostly automatic) before starting the filtration of the daily batch. They generally use raw groundwater to backwash the sand filter and AC filter. The softener unit is regenerated by injecting a normal salt (NaCl) solution into the resin bed. In some other RO systems, the backwashing interval varies in between 2 days to 1 month. A few RO stations were reported as not following proper backwashing practices. Altitudes of the operators towards this service directly influence this kind of issue, which leads to a reduction overall RO system life span and weakening the economy of the CBOs.

Normally, the sediment filter cartridge (MF, UF) in the pre-filter is replaced by the operator. The requirement of replacing that filter depends on the knowledge of the operator and training they have taken. In most cases, sediment cartridge replacement intervals were fixed. A total of 33.8% of CBO-established RO stations would replace their cartridge filters once every three months. This has been fixed by NWSDB or NGOs who consult with the CBOs. A few of the RO stations (<5) have been recorded as never replacing the sediment cartridge/MF after installing the station. In the majority of RO systems under CBOs, this pre-filter was usually dissembled, washed, and cleaned using treated water once a week to reduce the replacement frequency and maintenance cost. A considerable number of RO operators (around 60% of RO stations) use color changes in the filtration membrane to indicate the replacement requirement. Usually, new filter cartridges (MF) are pure white, and after few days of operation, they become dark (brown or black) in color due to clogging sediments and inorganic precipitants such as oxides of iron and manganese in the feed groundwater. Frequent cleaning becomes ineffective after a few cleaning sessions, indicating the need for replacement of the filter cartridge. Generally, replacement intervals of sediment cartridges vary from 1 week to 6 months, depending upon the feed groundwater quality and the efficiency of the sand/activated carbon filters and softening unit.

Generally, RO membrane elements are replaced after a few years of operation due to the fouling and scaling, which leads to a reduction of water recovery. Replacement frequency is governed by maintenance and operational practices, the efficiency of the pre-treatment system, and the feedwater quality. In the NCP, the majority of the CBO-operated RO stations replace their membrane elements with a 2–3 year interval (Figure S5). Regular monitoring filtration performances are carried out by relevant consulting organizations and decide the required time to replace the membranes. Lower permeate recovery with the elevated system operating pressure due to the fouling or scaling is the indicator of the requirement of replacing the membrane elements. Normally, removed RO membranes are not regenerated to reuse. Therefore, new RO membrane elements have to be purchased by the CBOs, and the cost is covered by the income of the CBOs.

Operating pressure is one of the major and critical parameters that indicate the health of the RO membrane. Maintaining the proper and standard pressure inside the membrane is quite challenging because it is directly influenced by the chemistry of the feed water [12]. The changes in actual operating pressures inside the pressure vessels are significantly influenced by RO feed water quality, as well as fouling/scaling. In this study area, investigated RO systems were operated under an average 54–192 psi (372–1324 kPa) pressure range inside the RO vessels. Exceeded pressure indicates severe fouling or scaling with reduction of the water recovery. For the pressure in the recommended range to be maintained, the concentrate flow rate is adjusted by the operator, and it changes the production rate of permeate water. Lower pressure below the standard range is an indication of a damaged membrane element. The membrane can be damaged due to overpressure exerted due to the scaling or fouling and oxidation by the residual chlorine or other oxidizing compound remaining in the pre-treated water [12].

### 3.3. Performance Analysis of RO Systems

#### 3.3.1. Water Quality

The quality of RO feed/source water (groundwater) and product water (permeate) is summarized as in the following tables (Table 1 and Table 2).

The majority of RO systems used groundwater as the primary feed water source. Slightly alkaline groundwater was observed in this region, in which pH value lied within the permissible range established by the WHO (6.5–8.5). A significant number of samples were observed as having elevated and unacceptable levels of EC (98%), hardness (17%), alkalinity (86%), fluoride (21.8%), and chloride (4%) in this groundwater while exceeding their MALs given by WHO and SLS, as summarized in Table 1. All the dominant constituents in the groundwater have an origin of geogenic minerals (calcite and silicate minerals) rich in the lithosphere in the NCP area [8,23,24]. Agricultural communities have been consuming this hard groundwater for several decades before discovering the CKDu in the dry zone of Sri Lanka [1,25]. Therefore, the primary objectives of the implemented RO technology in this region were to remove the excess minerals to produce low mineralized, palatable, and toxin-free drinking water [10,15]. It was observed that 100% of samples were had water quality below the MALs established by relevant authorities (Table 2). The removal rates of dissolved constituents in the groundwater by this RO technology were significantly higher compared to the conventional methods. Some studies have emphasized that RO product water in these regions have unacceptably lower levels of essential minerals such as calcium, magnesium, and fluoride [10,15]. According to the results (Table 1 and Table 2), the trace elements including Mn^2+^, Fe^2+^, Cd^2+^, As^3+^, Cu^2+^, Zn^2+^, Cr^3+^, and Hg^2+^ were observed within the acceptable levels in both groundwater and RO-treated water. In addition, the lack of fluoride in the permeate was one of the key findings. The groundwater in this study area (21.8%) has an unacceptable level of fluoride levels (>1.5 mg/L), which has led to severe health issues such as dental and skeletal fluorosis among the community in this area [26,27]. The fluoride removal efficiency (96.6%) was significantly efficient by this treatment technology, but 95% of the RO drinking water samples were below the minimum permissible limit (<0.5 mg/L) by WHO and SLS standards. Therefore, most RO systems produced drinking water with higher quality that are safer and palatable for human consumption while mitigating the low essential minerals by adding minerals externally. However, previous studies have reported that vegetables and fruits that were cultivated in the dry zone region contain more minerals (F^−^, Ca^2+^, and Mg^2+^) due to geological minerals in the soil and irrigation water [28]. It is obvious that the crops cultivated in these mineral-rich soils will add more essential minerals into the consumers’ systems through their diet. Thus, lack of minerals in drinking water could not be a significant issue for communities in the study area. Previous studies recommended implementing advanced purification methods such as NF and EDR on the basis of the reported lower rejection rates of essential minerals [20,29,30]. The major drawback of these technologies are expensive and do not perform efficiently for brackish water treatment in Sri Lanka as much as expected [15]. Thus, few economically feasible solutions can be recommended by this study. Mixing RO permeates with pre-treated groundwater and surface water would be a simple solution rather than going for expansive and advanced treatment process implementations. Another solution is that the community should be well aware of this issue and encourage them to fulfil their nutrient need with other sources such as fruits and vegetables that are rich in minerals in the dry zone of Sri Lanka.

#### 3.3.2. Comparison of RO Performances

CBO-operated RO stations showed different performance levels and efficiencies due to deviations in feedwater quality, system configurations, pre-treatment processes, and operating and maintenance practices. Major performance indicators of RO systems such as permeate water recovery, salt rejection, individual ion rejections, membrane selectivity, and permeability for different constituents were evaluated in this study.

##### Permeate Recovery

**Figure 2:**
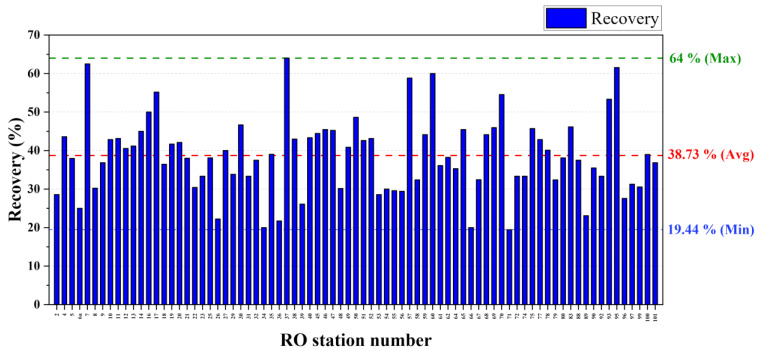
Permeate recovery rates of each RO stations in NCP.

Permeate water recovery was a major concern in the performance of membrane-based water treatment stations. It affects the energy efficiency as well as the purification efficiency of the system. Thus, maintaining higher recovery rates reduces cost of operations. Exceeding the recommended recovery value can lead to serious membrane fouling and scaling, which could reduce the membrane lifetime. Having significantly lower recovery rates was one of the major issues within the RO systems in the NCP. Figure 2 and Figure S6 show the detailed permeate recoveries in each drinking water station.

The average water recovery was around 38.7%, and the majority of the RO stations (89%) showed below 50% of water recovery. Further, the concentrate was more than 50% of the feed water. Generally, the recovery of the RO system is decided by the designer. It is a fixed value in the range from 50% to 85%, and generally it is designed to achieve 75% water recovery [12]. Exceeding this range results in a high fouling and scaling rate due to lack of water flow in the concentrate side of the membrane, and reducing the permeate water quality and lowering the recovery results in higher production of wastewater, which leads to the lower energy efficiency of the system. As shown in Figure 2 and Figure S6, it can be realized that a considerable amount of energy has been wasted by producing an unnecessary amount of wastewater. Most of the RO stations in this study area maintain their operating pressure at a fixed value, which was recommended by the consulting organizations, and it was usually adjusted by changing the concentrate flow by a manual valve. Gradual fouling and scaling on the membrane under fixed constant pressure operation leads to lowering of the permeate flow and increase in the concentrate flow due to reduction of the water permeability of the membrane. As a result, lower permeate recovery can be observed in older membranes. The effort to obtain higher recovery from these fouled membranes by lowering the concentrate flow could lead to an increase in the operating pressure at an unacceptable level. It can be led to damage to the membrane element and the high-pressure pump. Lower recovery and higher operating pressure are indications of RO membrane fouling and scaling. Hence, this indication was frequently used to decide the requirement for membrane replacements.

RO systems in the NCP consisted of small number of RO elements such as one (22%), two (44%), or three (26%) elements per RO system (Table S2). Usually, a single RO membrane element can achieve only around 15% recovery under the standard operating pressures (Table S3). Thus, the maximum recovery it can achieve is 45% (15% recovery per element × 3 elements) for most RO systems. Therefore, having lower recoveries from these RO systems is obvious.

In this investigation, we identified and revealed that most RO systems are operated under much lower operating pressures while ignoring the recommendations of the manufacturer. Figure S7 illustrates the standard pressure values and actual operating pressures of selected RO systems. Usually, RO membranes used in the NCP can be categorized into three groups on the basis of their operational pressure ranges. They were low pressure (LP: 225 psi or 1551 kPa), ultra-low pressure (ULP: 150 psi or 1034 kPa), and extremely low pressure (XLP: 100 psi or 690 kPa). According to the pressure and performance data, we found that lower recovery could occur due to lower operating pressures as well. Therefore, the operators should be well educated and trained for proper maintenance and standard operating conditions for the RO systems to achieve optimum productivity while increasing the durability and lifespan of the system.

##### Hardness and Alkalinity Rejection

Higher hardness level in the groundwater was common in this region—83% of the wells consisted of hard (120–180 mg/L CaCO_3_) and very hard (>180 mg/L CaCO_3_) groundwater in NCP. Unpalatable nature with unpleasant taste was the major issue with the higher hard waters [8,31]. In some studies, the total hardness of the drinking water was also examined for the CKDu cause [6,15,32]. Feedwater total hardness is an important factor to be addressed by this technology to produce drinking water. That was one of the major expectations when introducing the RO technology to the NCP last decade. This study observed relatively higher hardness removal rates in the existing RO systems in the NCP. The hardness rejection varied from 66.2% to 99.6% while having a significantly higher average of 95.8% (Figure S8). The results showed RO membranes have a higher capacity to demineralize hard water. Even though drinking water quality standards have not defined lower limits for essential minerals, some studies have suspected that long-term consumption of demineralized water leads to deficiencies in essential elements such as calcium and magnesium and causes serious health effects [13,14]. Re-mineralization of permeate can be achieved by filtering through a calcite bed/contactor [33,34] or mixing with pre-treated sediment and pathogenic bacteria-free source water.

**Figure 3:**
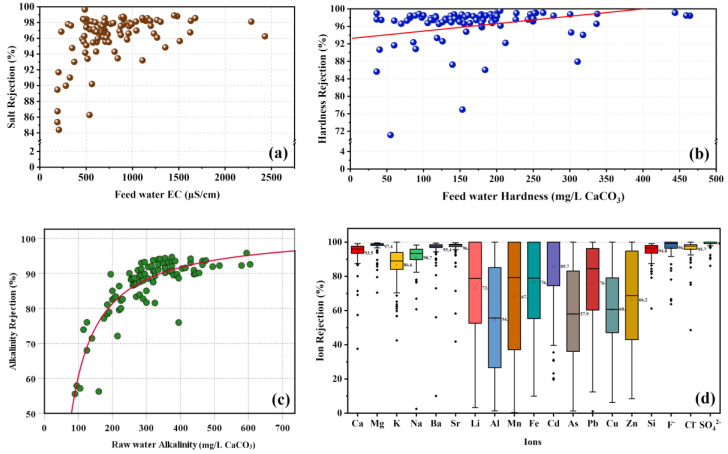
Performance of RO stations: (**a**) the salt rejection variation with the feed water EC; (**b**) hardness rejection efficiency with the feed water hardness; (**c**) alkalinity rejection rates with corresponding feed water alkalinity; (**d**) rejection of individual ions and silicon.

Deviation of hardness rejection depends on the interaction of Ca^2+^ and Mg^2+^ ions with the RO membrane surface. The surface chemistry of the polyamide thin film composite (TFC) RO membranes and the feedwater quality control the rejection mechanism. Therefore, rejection of hardness can be explained by multivalent ion rejections by the RO membranes. Results showed that hardness rejection was significantly higher when feeding very hard groundwater to the RO systems (Figure 3b). The reason is the creation of ionic bonds between negatively charged polyamide TFC membrane surface (carboxyl and hydroxyl groups) and positively charged cations [35,36,37]. This phenomenon further enhances the rejection of multivalent ions but it encourages the fouling and scaling tendency on the membranes. Scaling by CaSO_4_ and MgSO_4_ can be accelerated due to the thickness of the stagnant water layer in the surface of the membrane, which is enhanced by the low water flowrate on the concentrate [12]. Hence, maintaining good recovery rates and concentrate flow upon the membrane is crucial to overcome the scaling by saturation of salts near the surface of the membranes. Similarly, RO systems with soft feedwater showed considerably lower hardness rejection for a significant number of RO systems (Figure 3b), which might have been due to the higher electrostatic attraction forces exerted by negatively charged membrane surface.

The alkalinity of groundwater is controlled by the dissolved inorganic carbon species (HCO_3_^−^ and CO_3_^2−^). Elevated levels of HCO_3_^−^-based alkalinity of groundwater in the NCP were generally indicated by their alkaline pH range (6.95–8.3). The rejection of alkalinity by RO systems varied from 55.6% to 96.9%, while having an average of 86.6% (Figure S9). Alkalinity rejection showed a moderate correlation with the feedwater alkalinity (r = +0.64, *p* < 0.01). Further, the rejection rate showed an increasing trend with the feedwater alkalinity (Figure 3c). This would have been due to the additional removal of HCO_3_^−^ ions that could occur at higher alkalinity by crystallization and precipitation near the membrane surface [38]. Usually, high alkaline groundwater consists of high hardness with Ca^2+^ and Mg^2+^ ions. The thicker concentration polarization (CP) layer occurs at high alkalinity and in very hard feed water, resulting in saturation limit reaching close to the membrane surface. Thus, crystallization of CaCO_3_, CaSO_4_, and MgCO_3_ can occur in the CP layer with high alkaline feed water. It further reduces the permeable HCO_3_^−^ ion content at the membrane surface while maintaining a lower ion flux through the polyamide matrix. In consequence, high alkaline feedwater shows higher rejection rates. However, CaCO_3_ scaling potential becomes relatively higher at these conditions. Similarly, at lower alkalinity, groundwater has lower inorganic ions. Hence, resistance to entering the TFC layer and migrating to the permeate side is significantly lower, except in terms of the slight repulsive forces exerted by the negatively charged polyamide surface functional groups while giving lower alkalinity rejections [39].

##### Salt Rejection

The salt rejection efficiency of a RO system is a crucial factor in evaluating its performance. Typically, RO membranes have relatively higher salt rejection capability (>99%) compared to other membrane filtration methods such as NF, UF, and microfiltration (MF) [12].

Salt rejection of 95 CBO-established RO systems was evaluated, except in six RO systems that could not be covered for collection of source water samples due to technical issues. The average salt rejection was 95.9%, in the range from 67.6% to 99.7%. The majority of CBO-established RO systems (93%) had salt rejection above 92% with excellent performance, except for seven RO systems (Figure S10).

According to the manufacturer, the minimum salt rejection would be above 99%, but results showed less salt rejection. Reasons might be improper operation and maintenance practices, degradation of the membrane with age, and membrane fouling effect [9]. At lower operating pressure, RO membrane shows comparatively lower salt rejection while increasing the rejection with the increasing pressure [29]. Table S3 shows the standard pressure values and relevant salt rejection rates of each type of membrane model. Generally, RO membrane manufacturers measure the rejection rates of their membranes using a known concentration of NaCl solutions (1500 or 2000 ppm) under the standard pressure values in order to achieve 15% permeate recovery per one RO element (Hydranautics/Nitto, Oceanside, CA, USA).

Figure 3d illustrates the individual ion rejections of the RO membranes used in drinking water stations in NCP. Most dominant metal cations in the groundwater, such as Ca^2+^, Mg^2+^, K^+^, Na^+^, Ba^2+^, Sr^2+^, and silicon (Si), were shown to have higher rejections at above 86%. Other metal cations, including trace metals such as Li^+^, Al^3+^, Mn^2+^, Fe^2+^, Cd^2+^, As^3+^, Cu^2+^, and Zn^2+^, showed comparatively lower average rejection values and higher standard deviations. However, an acceptable level of trace metals in the product water was observed.

Most dominant anions in the groundwater were Cl^−^ and SO_4_^2−^, while the availability varied in the order of Cl^−^ > SO_4_^2−^ > NO_3_^−^ > F^−^ > Br^−^. The rejection of dominant anions was analyzed to understand the performance of the filtering process by RO systems. The results showed that RO membranes had a higher capability of removing of dominant anions (>94%) in the groundwater to produce safe drinking water with lesser inorganics.

##### Factors of Ion Rejection

Twenty-one units of RO systems (Pure Aqua, Santa Ana, CA, USA) with the same RO membrane ESPA2-LD-4040 manufactured by Hydranautics were analyzed for correlations with different operational factors such as feedwater EC, individual ion concentrations, operation pressure, and membrane age (Table 3 and Table S4).

**Figure 4:**
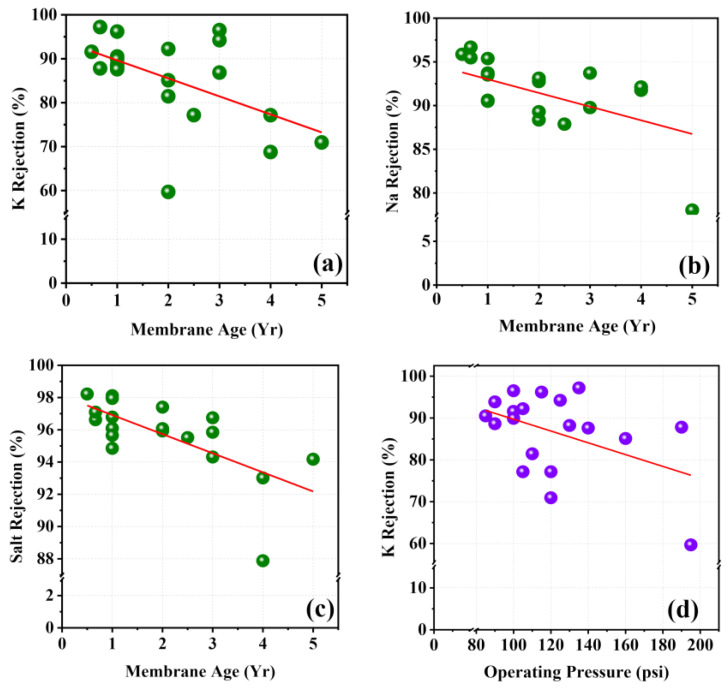
Rejections of monovalent ions with membrane age: (**a**) K rejection, (**b**) Na rejection, (**c**) salt rejection with membrane age, and (**d**) K rejection with operating pressure.

Membrane age is an important parameter that influences ion rejection. Usually, the membrane replacement interval varies from 6 months to 3 years in the NCP RO stations. Older membranes showed a significant reduction of rejection compared to newer membranes. The rejection of major cations in the feed water such as Ca^2+^, Mg^2+^, K^+^, Li^+^, and Na^+^ showed low-to-moderate negative Pearson correlations (−0.42, −0.442, −0.550, −0.563, and −0.456: *p* < 0.05) with the membrane age, with an indication of the degradation of the membrane with the time (Table 3). K^+^, Li^+^, and Na^+^ ions showed a significant relationship with the membrane age (Figure 4a,b). This may have been due to the lower hydration radius, higher mobility of the monovalent ions, and distorted degraded TFC layer [40]. Similarly, salt rejection also showed a significantly higher negative correlation (r = −0.68, *p* < 0.01) with the membrane age and reduced rejection capability (Figure 4c).

Monovalent ions such as K^+^ showed a moderate negative correlation (r = −0.41, *p* = 0.062) with the operating pressure, while other ions showed a very weak negative correlation (Figure 4d). Smaller hydration radius and higher mobility of the monovalent ions gave comparatively lower resistance for intrapore diffusion through the polyamide matrix [39]. Similarly, slight expansion (swelling) of polymer (polyamide) matrix on the TFC layer at higher pressure could lead to increase in the porosity, allowing for permeation of more ions with smaller hydration radius, such as K^+^, through the membrane [41,42]. Such phenomena could be the major reason for having lower rejection of K^+^ ions at higher operating pressure, as Figure 4d shows.

**Figure 5:**
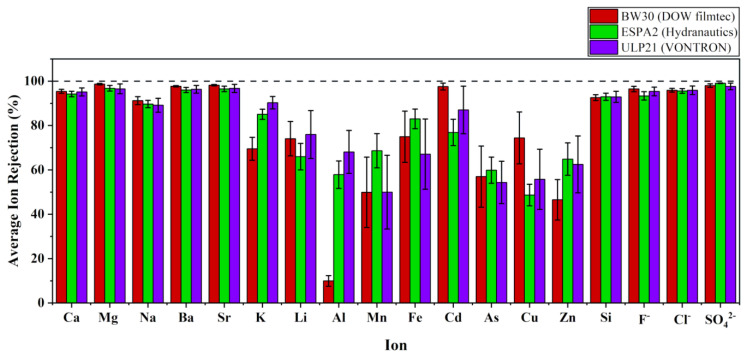
Average rejection of individual ions by three types of RO membranes.

All the RO membrane modules used in RO systems in NCP were polyamide TFC membranes. The most common products were DOW filmtec, Hydranautics, and VONTRON (Table S5). For the rejection of individual cations, these different RO elements showed different values for each cation. Figure 5 shows the number of RO stations that were used to determine the average rejection rates in this analysis.

Apart from the higher level of rejections (>89%), almost similar performances from all three types of membranes (BW30, ESPA2, and ULP21) were observed for dominant constituents (Ca^2+^, Mg^2+^, Ba^2+^, Sr^2+^, Na^+^, Si, F^−^, Cl^−^, and SO_4_^2−^). However, the rejection of monovalent cations (Li^+^, K^+^) and other trace metals (Al^3+^, Mn^2+^, Fe^2+^, Cd^2+^, As^3+^, Cu^2+^, and Zn^2+^) showed different average values for each type of RO membrane. The lowest average rejection was observed for Al^3+^ ions by BW30 membrane, which was around 10% rejection. However, for Fe^2+^, Cd^2+^, and Cu^2+^, BW30 membrane showed comparatively higher rejection performances. Generally, for heavy metal removal, ESPA2 and ULP21 membranes showed sufficiently good performances.

##### Variation of Performances with Different RO Configurations

**Figure 6:**
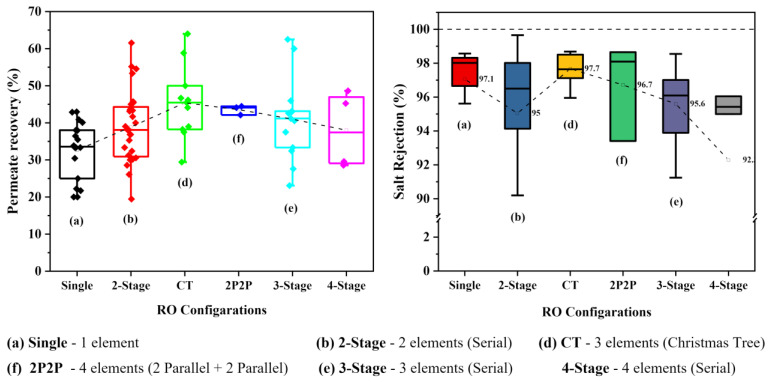
Variation of permeate recovery (**left**) and the salt rejection rates (**right**) with the common RO configurations in NCP. Configurations indicated by (**a**,**b**) and (**d**–**f**) are illustrated in Figure S2.

Figure 6 shows the comparison of performance in RO systems on the basis of their RO element configuration/arrangement. Multi-stage RO systems showed significantly higher water recovery compared to single-stage systems. However, double-stage RO systems with Christmas tree arrangement (CT) recorded the optimum average recovery (45.4 ± 9.8%) value. Similarly, the salt rejection rate by the CT configuration also reported the highest value (97.7 ± 0.85%) among the different configurations while showing the best performance status, suggesting its suitability for sustainable water purification. Even though lower recovery values were reported with the RO systems with lower number of RO elements, salt rejection rates did not show a significant relationship with the number of stages or RO elements.

##### Ion Selectivity and Permeability of Polyamide TFC RO Membranes

Ion selectivity and permeability of RO membranes are significantly important factors that affect the product water quality. The surface chemistry of the membrane and the feedwater quality are the major factors that control the ion selectivity and permeability. Thus, different relationships between ionic constituents in the feedwater and the permeate were compared to understand the mechanism behind the deviation of product water quality and the influence of membrane surfaces of RO systems.

- Selectivity and permeability of cations

**Figure 7:**
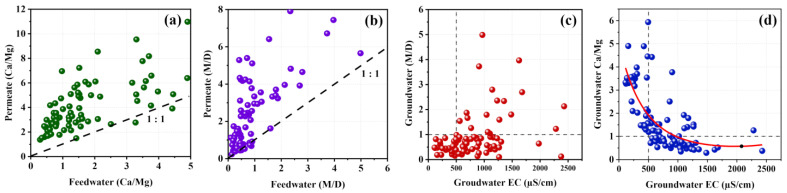
(**a**) Ca/Mg selectivity and permeability; (**b**) cation selectivity of monovalent/divalent (M/D) RO TFC membranes and (**c**) M/D ratio with groundwater EC; (**d**) Ca/Mg ratio with groundwater EC in NCP.

Figure 7 illustrates the relationship of major cations in permeate against the feedwater. Figure 7a shows that almost all the Ca/Mg fractions of the permeate were positioned above the 1:1 line. Thus, the Ca/Mg fraction was increased in the permeate compared to the feedwater, indicating the significantly higher permeability of Ca^2+^ ions in comparison with Mg^2+^ ions through the RO membrane. It confirmed the higher selectivity of Ca^2+^ ions for polyamide RO membranes.

Monovalent to divalent cation fraction (M/D) was increased after RO filtration (Figure 7b), revealing that permeate contains a higher monovalent cation percentage compared to the raw feed water. It indicates that the permeability of monovalent ions through polyamide RO membranes was higher than the divalent ions. The polyamide TFC layer allows for a considerable number of monovalent ions to pass through while providing significantly higher resistance to divalent hardness metals due to the lower hydration radius and higher mobility of monovalent ions [43].

As shown in Figure 7c, groundwater with a lower level of EC (<500 µS/cm) showed a significantly lower M/D ratio (<1), indicating the dominance of divalent ions in this region. However, high salinity groundwater seemed to have a significantly higher M/D ratio due to the presence of dominant monovalent cations in comparison with divalent cations. Similarly, at a lower level (<500 µS/cm) of salinity (EC), Ca/Mg ratio was comparatively higher (>1), and groundwater with a higher level of salinity showed a lower Ca/Mg ratio, which was an indication of Ca^2+^ dominancy in low salinity groundwater and Mg^2+^ dominancy in high salinity groundwater (Figure 7d).

**Figure 8:**
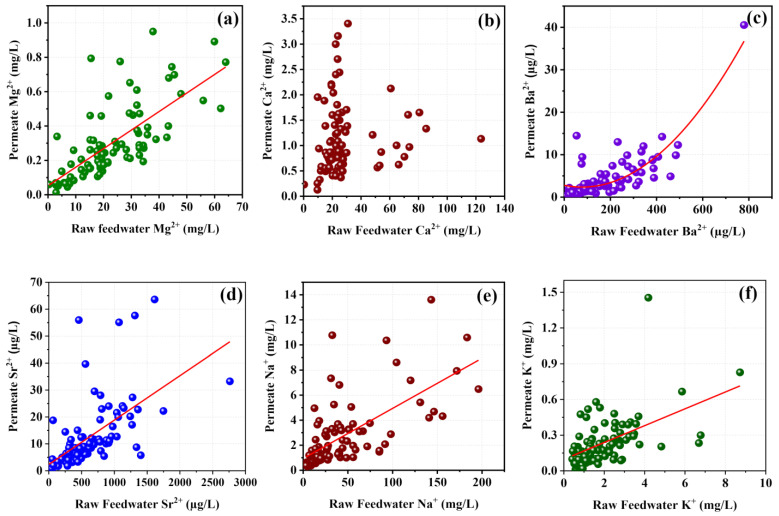
Major cation concentrations in the permeate with their feedwater cation concentrations, (**a**) variation of Mg^2+^ in the permeate with Mg^2+^ in the feedwater, (**b**) variation of Ca^2+^ in the permeate with Ca^2+^ in the feedwater, (**c**) variation of Ba^2+^ in the permeate with Ba^2+^ in the feedwater, (**d**) variation of Sr^2+^ in the permeate with Sr^2+^ in the feedwater, (**e**) variation of Na^+^ in the permeate with Na^+^ in the feedwater, and (f) variation of K^+^ in the permeate with K^+^ in the feedwater.

Ca^2+^ ions, as the most dominant in groundwater, have significantly higher permeability through polyamide TFC membranes. When they are in lower concentrations, permeate Ca^2+^ ions tend to increase with the feedwater Ca^2+^ ion concentration (Figure 8b). This is because the electrostatic repulsion forces exerted by attached divalent ions (Ca^2+^ and Mg^2+^) to the functional groups on the surfaces of the membrane is lower when feedwater contains dilute level of Ca^2+^ ions. The only resistance that affected the molecular transport of calcium is the energy barrier of the intrapore diffusion [39,44], resulting from the cations’ interaction with the negatively charged internal pore walls. When the ion concentration becomes higher (especially Ca^2+^), more divalent ions attach to the membrane surface and cover a significant portion of the membrane surface [36] while providing enough repulsive force on the cations in solution. This restricts the entrance of calcium ions into the polyamide layer while resulting in a lower and stable calcium ion level in the permeate.

Even though Mg^2+^ ions have a higher level in feed, they have a comparatively higher hydration radius due to the higher charge density, which lowers the mobility and permeability in the solution [43,45]. Therefore, it reduces the capability of moving (permeability) through the polyamide matrix in the TFC layer, and permeate Mg^2+^ ion content keeps below 1 mg/L while following the linear relationship with the feed water Mg^2+^ ion level (Figure 8a). The interaction and binding energy of metal ions with the membrane surface group also controls the ion flux through the TFC layer. Usually, for carboxylic oxygen on polyamide membrane surfaces, divalent ions (Ca^2+^ and Mg^2+^) have higher binding energy than monovalent ions, while the potential of bonding (attachments to the surface functional groups) order is maintained as K^+^ < Na^+^ < Ca^2+^ < Mg^2+^ [46]. Therefore, a comparatively higher fraction of Mg^2+^ ions is strictly attached to the polyamide surface while a large portion of Mg^2+^ is ions rejected with the concentrate flow. Other cations that have lower levels in the feed water showed closely linear increasing trends with the feedwater ionic level, resulting in a lower level of these ions in the permeate (Figure 8c–f).

However, some monovalent ions such as sodium, present in higher concentrations in the feed, showed an increasing linear trend with the feed without being affected by the valance (Figure 8e). This may have been due to the higher permeability of monovalent ions in comparison with divalent ions. Monovalent ions have higher permeability through polymer membrane due to their lower hydration radius and higher mobility in aqua solution [43,47].

II Permeability of anions

**Figure 9:**
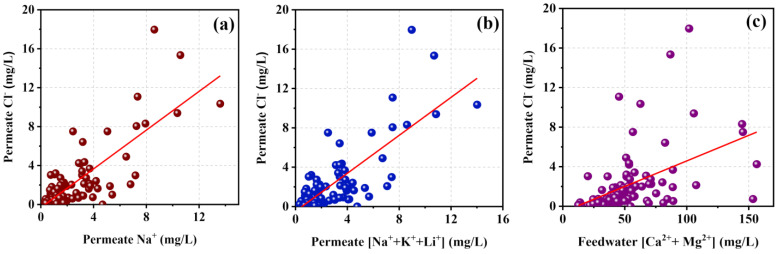
Chloride permeability through RO membranes with the concentration of (**a**) Na^+^ in the permeate, (**b**) total monovalent cations in the permeate, and (**c**) divalent cations in the feedwater.

The surface of the polyamide TFC membranes used in RO systems in this study area was negatively charged due to the higher pH level of feed groundwater [48,49]. The electrostatic repulsion forces exerted by the surface charge on the anions were significantly higher. This phenomenon enhanced the rejection of anions, providing higher resistance to the partitioning of anions into the membrane matrix [39]. However, with the higher concentration of multivalent cations in the feedwater, major anions such as Cl^−^ showed improved permeability through the RO membranes (r = +0.727. *p* < 0.01) (Figure 9c). This occurred because of the most dominant divalent cations such as Ca^2+^ and Mg^2+^ ions in the feed, covering the negatively charged membrane surface by attaching to the surface functional groups while reducing the negativity of the surface, weakening the electrostatic repulsion forces on anions. Similarly, permeable Cl^−^ ions showed strong positive correlation with the permeable monovalent cations, especially for Na^+^ (r = +0.71. *p* < 0.01) (Figure 9a,b). This can be explained by interactions among monovalent cations, major anions, and pore walls of the membranes. Two major mechanisms can explain the anion diffusion through the polyamide membranes. The first one is anion transfer by pairing with the permeable cations to maintain the ion charge balance of the permeate [39,44,47]. The second mechanism is the separate permeation of anion due to the governing force of membrane potential exerted due to the fast permeation of monovalent cations with lower resistance to diffuse [50,51]. This membrane potential drags and speeds up the major anion (Cl^−^) movement through the TFC layer.

Silicon in the groundwater is usually neutral and stable (Si (OH)_4_) at neutral and slightly acidic conditions. At higher alkalinity or higher pH, some fraction is converted into HSiO_4_^−^, which has an ionic nature [52]. Consequently, the driving potential exerted by fast-moving cations (monovalent) and its pairing effect, HSiO_4_^−^ transfer, was increased through the TFC layer, which was explained by the positive correlation between permeate monovalent cation level (especially Na^+^) and silicon level (r = +0.531. *p* < 0.01).

As usual, the concentration of ions in the permeate was directly affected by the feed water ion concentration. Thus, dominant Cl^−^ and SO_4_^2−^ ions showed moderate and strong positive correlations with their feed concentrations, respectively (r = + 0.685 and +0.952, *p* < 0.01, respectively).

### 3.4. Cost Analysis

Table S1 shows the product water price ranges of RO stations operated by the relevant organization. The majority of the villagers belong to low- or middle-income populations. To develop health conditions and lifestyle, CBOs supply the RO drinking water at a reasonably low rate. Most of the CBO-operated RO stations (84% of RO stations) provide product drinking water at the standard price of LKR 1 (Sri Lankan rupee) per one liter. It ranges from 50 cents per liter to LKR 1.5 per liter. Usually, the average monthly income of the CBO-operated RO stations was around LKR 54,062.00, with the range from LKR 2250.00 to LKR 210,000.00 (Figure S11). Even though a few CBO-operated RO stations try to spread their services well, many RO stations were not well maintained. This depended on the attitude of the operators and CBO management, knowledge of the operators, and willingness to develop their service to the public community.

The average production cost of drinking water was estimated for a typical RO station in the NCP (Table S6), considering the cost for membrane replacements, antiscalants, replacements of pre-treatment materials (sand, AC, resin, sediment cartridges), pump repair, electricity, and salaries of the operators. Every material price was estimated according to the current rates in the Sri Lankan market. RO membrane elements, pre-filter materials, and sediment cartridge replacement intervals were assumed as two years, two years, and one month, respectively, on the basis of averaged values recorded in this investigation. The calculated average production cost of product water was around 49 cents per one liter (LKR 0.49 /L) in typical RO drinking water stations in the NCP that operated an average of 4.4 h/day. When it was assumed that the operational time was 20 h and working in full capacity (10,000 L/day), we found that production costs were significantly lower at around 15 cents per liter (0.145 LKR/L). Therefore, the results indicated that working in full capacity can reduce the production cost of drinking water.

## 4. Challenges and Recommendations

### 4.1. Water Quality Issues

Even though lower limits of water quality standards for total mineral content (EC or TDS) in the drinking water have not been clearly defined, consumption of demineralized water may lead to serious health issues. Similarly, insufficient fluoride levels (<0.5 mg/L) in the product water were also observed in this study due to the very high rejection capability of these RO membranes. Unnecessary levels of higher rejection rates for essential minerals are the major drawback of RO technology. Thus, increasing the level of essential minerals in RO product water remineralization is necessary. Major remineralization processes that RO stations used in the NCP are filtering through a calcite contactor and dosing a mineral solution to the product water. As mentioned in the previous sections, economically feasible simple methods such as mixing permeate with pretreated raw groundwater can be used to raise the mineral level in the product water. However, daily diet (fruits and vegetables) can fulfil the essential mineral need of the communities in the dry zone.

Even though the majority of villagers do not have proper knowledge about the EC value or TDS value, they have the uncertainty of whether RO drinking water has a low mineral level. This was one of the reasons for refusing to consume RO drinking water by some fraction of people in the study area. Therefore, frequent water quality monitoring should be carried out. In extreme and critical cases, alternative technologies such as NF and EDR, which have a higher selectivity for essential minerals to produce safer drinking water, should be checked for feasibility and implemented.

### 4.2. CBO Organizational Issues

CBO-operated RO stations have great competition with other organizations such as the Sri Lankan Navy and private commercial organization-operated RO stations. This competition greatly affects the quality of the service they provide. Sri Lankan Navy-operated RO stations supply their RO drinking water free of charge. Consequently, villagers tend to take the water from them. Nearly 817 of navy-operated RO stations are already established throughout the country [11]. Normally, in the majority of villages in the NCP, at least one navy-operated RO station is available. Thus, demand for the CBO-operated RO water has declined. Consequently, the economy of the CBOs becomes weaker, and lower-income leads to difficulties in terms of handling the maintenance works. Thus, managing the salaries of operators as well as operational and maintenance costs is becoming a major issue for several RO stations under CBOs. Some of the CBO-established RO stations have already been shut down permanently due to this economic issue.

Private company-operated RO stations are selling their water at significantly higher rates than the CBOs. However, they have home water delivery services. It is very convenient for the villagers, even though the price is higher. Therefore, especially in urban regions, this type of RO water service is more popular than CBOs. To expand their service, CBO RO stations should be focused on starting delivery services and installing drinking water storage tanks in different parts of the villages for the convenience of the community. There are a few CBO RO stations already distributing drinking water using these kinds of water tanks. This helps to increase consumers and develop the economy of the CBOs as well as rural community well-being.

In some of the areas in the NCP, the majority of the community depends on pipe-borne drinking water (purified tank water). Some of the areas with national reserves are rich in high-quality spring water. The majority of the villagers depend on spring water distribution by the government using bowsers. Hence, these types of RO stations are not common, and some RO stations have already been shut down due to the lack of consumers. Therefore, it is crucial that regular water quality monitoring and surveying of the water demand occurs in rural regions before advanced water purification technologies are installed.

For the quality of the service to be improved, the CBO-established RO station network should be well organized, and continuous monitorization of product water quality should be carried out. To improve the altitudes toward this social service, RO system operators and other responsible persons should be well trained and educated by the relevant organizations. This would improve the operational and maintenance practices of the RO stations and drastically reduce the cost for the operation and maintenance, increasing the lifespan of the RO systems. Villagers should be well educated about the importance of consuming safe drinking water and how RO drinking water helps to improve their health.

## 5. Conclusions

Lack of essential minerals in the product drinking water, lower recovery rates, fouling and scaling, and lack of proper and standard guidelines for RO system operation have been identified as major issues with RO stations in the NCP.

Source water had elevated and unacceptable levels of EC (98%), hardness (17%), alkalinity (86%), fluoride (21.8%), and chloride (4%) while exceeding their MALs given by WHO and SLS drinking water guidelines. Treated RO drinking water was well below the MALs. However, 95% of samples had an unacceptable lower level of fluoride (<0.5 mg/L), leading to increased dental caries. Simple and economically friendly methods such as mixing permeate with pre-treated groundwater were suggested to raise essential minerals (fluoride, calcium, and magnesium), and daily diet was suggested in order to fulfil the nutrient need of the consumers.

Having insufficient numbers of RO elements in each system in the NCP, operation under lower pressure, and membrane fouling were the major causes for lower permeate recoveries (average, 39%), which ranged from 19.4% to 64%. The majority (>93%) of RO systems showed higher salt rejection rates (>92%). Excellent removal rates of hardness and alkalinity were averaged at 95.8% and 86.6%, respectively. However, Christmas tree RO configuration was identified as the best option for sustainable water purification, which showed optimum recovery and salt rejection rates. Most dominant ions in groundwater such as Ca^2+^, Mg^2+^, K^+^, Na^+^, Ba^2+^, Sr^2+^ Cl^−^, F^−^, SO_4_^2−^, and silicon (Si) were shown at higher rejections and were averaged at 93.5%, 97.4%, 86.6%, 90.8%, 95.4%, 96.3%, 95.7%, 96.6%, 99.0%, and 94.8%, respectively. The trace elements such as Mn^2+^, Fe^2+^, Cd^2+^, As^3+^, Cu^2+^, and Zn^2+^ showed significantly lower rejections. Rejection of monovalent ions (Na^+^, K^+^) showed a decreasing trend with the membrane age and operating pressure due to degradation of polyamide matrix of the TFC layer and membrane swelling at higher pressures.

The permeability of monovalent ions was significantly higher than divalent ions through polyamide RO membranes, while significantly higher Ca^2+^/Mg^2+^ selectivity was observed. Permeability of Cl^−^ ion was improved with the concentrations of multivalent ions (Ca^2+^ and Mg^2+^) in the feedwater and permeable monovalent cations (Na^+^).

Most dominant constituents (Ca, Mg, Sr, Ba, Na, Si, F, Cl, and SO_4_^2−^) present in the source water showed a higher and similar level of removal efficiencies (>89%) for all three types of membranes (BW30, ULP21, and ESPA2).

The estimated production cost of RO drinking water was 49 cents (LKR 0.49/L) for a CBO-operated RO station with an average operational duration of 4.4 h. However, full-time-operated (20 h/day) RO stations showed 15 cents (LKR 0.145/L) of production cost while indicating that lower operational duration increases RO drinking water production cost.

Lack of knowledge and lack of proper guidelines for operation and maintenance practices leads to degradation of performance of ROs. Thus, CBOs may require further assistance from the water supply authorities regarding chemical cleaning of the RO systems, water quality testing, and technical training for operators.

## Figures and Tables

**Table 1:** Feedwater (groundwater) quality of stations in NCP with the comparison of drinking water quality standards (WHO and SLS).

Parameter	Units	Average	Max	Min	WHO MALs	SLS MALs	Unacceptable Samples (%)
Feedwater (groundwater)							
General parameters							
pH		7.1	8.3	6.95	6.5–8.5	6.5–8.5	0
EC	µS/cm	770	2430	112	400	750	98
Hardness	mg/L (CaCO_3_)	179.5	814	2.42	-	250	17
Alkalinity	mg/L (CaCO_3_)	320.4	1120	90	-	200	86
Anions							
F^−^	mg/L	1.16	4.9	0.03	1.5	1.0	21.8
Cl^−^	mg/L	78.9	473.6	0.29	-	250	4
Br^−^	mg/L	0.44	1.6	0.08	2	-	0
NO_3_^−^	mg/L	1.82	14.8	0.03	50	50	0
PO_4_^3−^	mg/L	4.08	6.3	0.93	-	-	0
SO_4_^2−^	mg/L	25.3	490.9	0.10	250	250	1
Cations							
Ca^2+^	mg/L	31.9	275.8	0.37	-	100	3.2
Mg^2+^	mg/L	23.94	64.01	0.37	-	30	33.7
Na^+^	mg/L	44.27	196.06	3.2	200	250	0
K^+^	mg/L	2.85	88.21	0.14	-	-	0
Li^+^	µg/L	7.47	26.65	0.354	-	-	0
Sr^2+^	µg/L	606.92	2763.05	2.960	-	-	0
Ba^2+^	µg/L	192.34	1698.17	0.340	1300	-	1
Mn^2+^	µg/L	0.72	25.86	0.057	100	100	0
Fe^2+^	µg/L	1.99	28.87	0.023	200	300	0
Cd^2+^	µg/L	0.2	0.59	0.010	3	3	0
As^3+^	µg/L	1.89	4.43	0.410	10	10	0
Cu^2+^	µg/L	2.17	14.27	0.055	2000	1000	0
Zn^2+^	µg/L	7.19	163.04	0.071	3000	-	0
Cr^3+^	µg/L	-	ND	ND	50	-	0
Hg^2+^	µg/L	-	ND	ND	6	-	0
Si	mg/L	45.03	95.95	3.690	-	-	0

**Table 2:** Product water quality from RO stations in NCP with the comparison of drinking water quality standards (WHO and SLS).

Parameter	Units	Average	Max	Min	WHO MALs	SLS MALs	Unacceptable Samples (%)
RO product (drinking) water							
General parameters							
pH		6.95	7.45	6.57	6.5–8.5	6.5–8.5	0
EC	µS/cm	27.6	153	1.7	400	750	0
Hardness	mg/L (CaCO_3_)	7.2	141.5	0.37	-	250	0
Alkalinity	mg/L (CaCO_3_)	35	95	0	-	200	0
Anions							
F^−^	mg/L	0.07	0.9	0.02	1.5	1.0	0
Cl^−^	mg/L	2.6	41.8	0.07	-	250	0
Br^−^	mg/L	0.14	0.15	0.13	2	-	0
NO_3_^−^	mg/L	0.31	1.5	0.10	50	50	0
PO_4_^3−^	mg/L	0.69	2.0	0.25	-	-	-
SO_4_^2−^	mg/L	0.58	14.8	0.08	250	250	0
Cations							
Ca^2+^	mg/L	1.684	20.82	0.126	-	100	0
Mg^2+^	mg/L	0.741	24.53	0.013	-	30	0
Na^+^	mg/L	3.164	51.65	0.310	200	250	0
K^+^	mg/L	0.292	3.01	0.011	-	-	-
Li^+^	µg/L	2.773	14.29	0.090	-	-	-
Sr^2+^	µg/L	17.37	380.85	0.378	-	-	-
Ba^2+^	µg/L	5.989	109.47	0.306	1300	-	0
Mn^2+^	µg/L	0.198	5.63	0.012	100	100	0
Fe^2+^	µg/L	0.938	9.32	0.010	200	300	0
Cd^2+^	µg/L	0.033	0.34	0.002	3	3	0
As^3+^	µg/L	0.796	2.37	0.031	10	10	0
Cu^2+^	µg/L	1.035	12.44	0.009	2000	1000	0
Zn^2+^	µg/L	4.453	107	0.011	3000	-	0
Cr^3+^	µg/L	-	ND	ND	50	-	0
Hg^2+^	µg/L	-	ND	ND	6	-	0
Si	mg/L	2.333	20.28	0.157	-	-	-

**Table 3:** Pearson correlation co-efficient of different operational parameters and individual ion rejections of ESPA2-LD-4040 membranes.

Parameter	Membrane Age	Operating Pressure	Feed Water EC
Salt rejection	−0.681 **	−0.026	0.182
Ca rejection	−0.421	−0.117	0.195
Mg rejection	−0.442 *	−0.049	0.008
K rejection	−0.550 **	−0.414	0.410
Li rejection	−0.563 *	0.360	−0.058
Na rejection	−0.456 *	0.060	0.264
As rejection	0.283	−0.229	−0.158
Si rejection	−0.396	0.132	0.198

** Correlation is significant at the 0.01 level (two-tailed); * correlation is significant at the 0.05 level (two-tailed).

## Data Availability

The data presented in this study are available on request from the corresponding author.

## Supplementary Materials

The following are available online at https://www.mdpi.com/article/10.3390/membranes11060383/s1, Figure S1. Process flow diagram of a typical RO system in the NCP. Figure S2. Different RO configurations that are common in NCP, Sri Lanka. Figure S3. Different options for reject water handling. Figure S4. Number of RO systems with their system age (Y). Figure S5. Percentage of the number of CBO-operated RO facilities (*x*-axis) in the NCP with their membrane replacement intervals (*x*-axis). Figure S6. Number of RO plants (*y*-axis) with the categorization by their water recovery as a percentage (*x*-axis). Figure S7. Tested (by manufacturer) operating pressure vs. actual operating pressure of selected RO systems in the NCP. Figure S8. Hardness rejection of each RO station. Figure S9. Alkalinity rejection rates of each RO station. Figure S10. Overall salt rejection for each RO plant (95 ROs) operated under CBOs (*x*-axis: RO plant number, *y*-axis: % salt rejection). Figure S11. Total income and total operating cost of each RO station in the NCP. Table S1. RO product water prices with their operating companies. Table S2. Diversity of RO system configurations in CBOs in the NCP. Table S3. Specification data of each RO membrane type used in RO stations in the NCP. Table S4. Influence of feedwater chemistry for ion rejection of ESPA2 membranes. Table S5. Selected RO membranes for comparison of individual ion rejection. Table S6. Estimation of production cost for drinking water in a typical RO station in NCP.
