# Supplementary material for: Evaluation of Performance of Existing RO Drinking Water Stations in the North Central Province, Sri Lanka
Source: Membranes (Basel). 2021 May 24;11(6):383. doi: 10.3390/membranes11060383 (PMC8225030; doi:10.3390/membranes11060383)
Supplement: Supplementary file 1 [file membranes-11-00383-s001.zip › membranes-1221162-supplementary.pdf]

membranes
MDPI

Supplementary Materials

Evaluation of Performance of Existing RO Drinking Water Stations in the North Central Province, Sri Lanka

Suresh Indika [1,2,3], Yuansong Wei [1,2,3,4,*], Dazhou Hu [1,2,3], Jegetheeswaran Ketharani [5], Tharindu Ritigala [1,2,3], Titus Cooray [6], M.A.C.K. Hansima [7], Madhubashini Makehelwala [8], K.B.S.N. Jinadasa [5], Sujithra K. Weragoda [9] and Rohan Weerasooriya [4]

1 State Key Joint Laboratory of Environmental Simulation and Pollution Control, Research Center for Eco-Environmental Sciences, Chinese Academy of Sciences, Beijing 100085, China; indika_st@rcees.ac.cn (S.I.); dzhu_st@rcees.ac.cn (D.H.); tharindu_st@rcees.ac.cn (T.R.)
2 Laboratory of Water Pollution Control Technology, Research Center for Eco-Environmental Sciences, Chinese Academy of Sciences, Beijing 100085, China
3 University of Chinese Academy of Sciences, Beijing 100049, China
4 National Institute of Fundamental Studies, Hanthana Road, Kandy 20000, Sri Lanka; rohan.we@nifs.ac.lk
5 Department of Civil Engineering, University of Peradeniya, Peradeniya 20400, Sri Lanka; ketha94@eng.pdn.ac.lk (J.K.); shamj@pdn.ac.lk (K.B.S.N.J.)
6 Department of Applied Earth Sciences, Uva Wellassa University, Badulla 90000, Sri Lanka; titus@uwu.ac.lk
7 Post Graduate Institute of Science (PGIS), University of Peradeniya, Peradeniya 20400, Sri Lanka; 127charithahansima@gmail.com
8 China-Sri Lanka Joint Research and Demonstration Center for Water Technology, Ministry of Water Supply, Meewathura, Peradeniya 20400, Sri Lanka; madhu10w@yahoo.com
9 National Water Supply and Drainage Board, Katugastota 20800, Sri Lanka; skwera7@gmail.com
* Correspondence: yswei@rcees.ac.cn; Tel.: +86-10-6284-9690

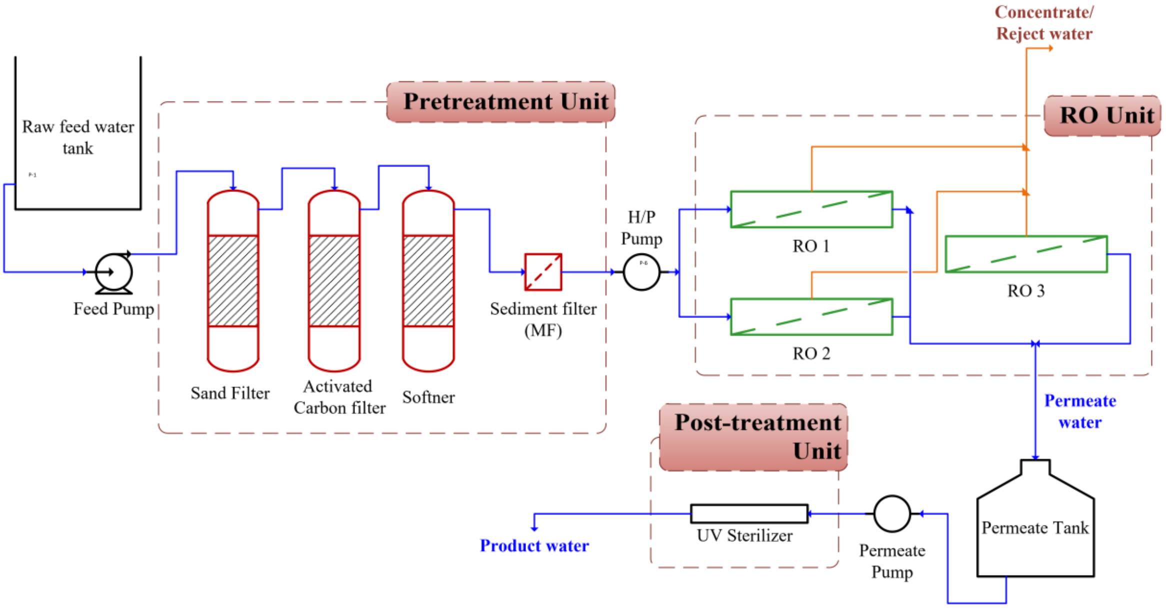

Figure S1. Process flow diagram of a typical RO system in the NCP

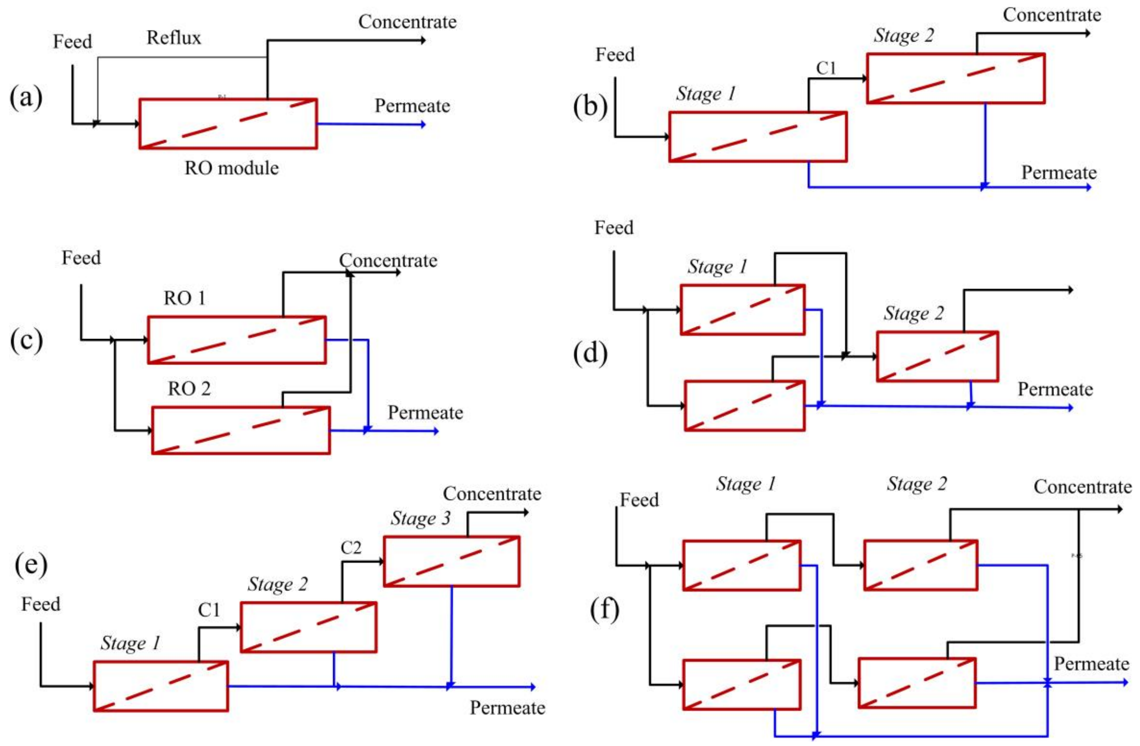

Figure S2. Different RO configurations that are common in NCP, Sri Lanka

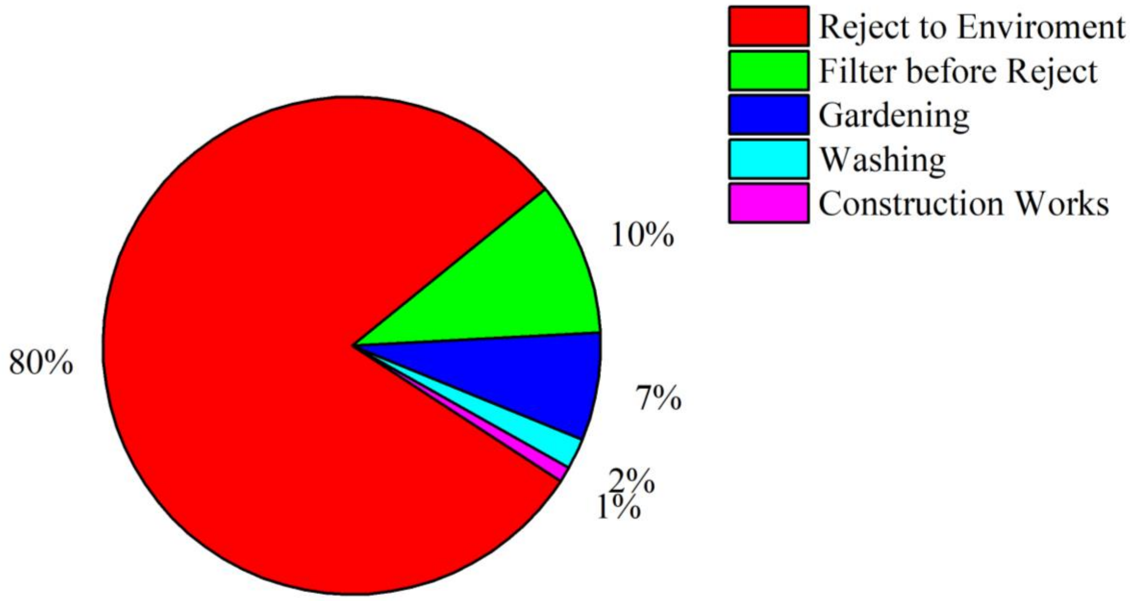

Figure S 3. Different options for reject water handling

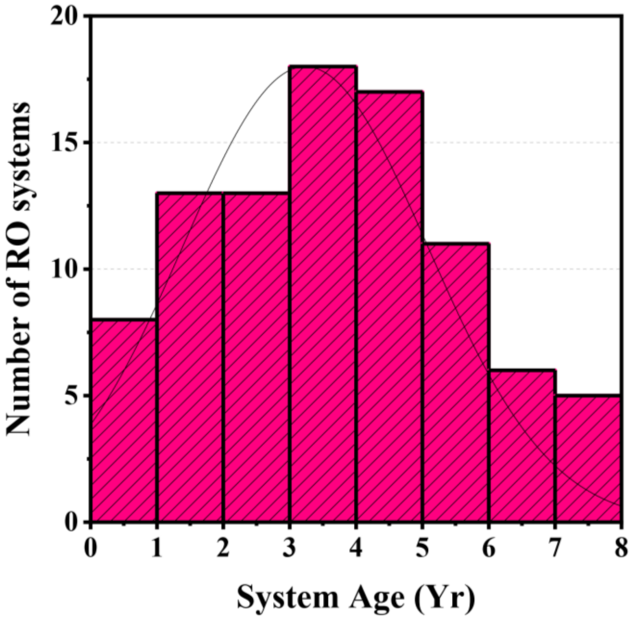

Figure S4. Number of RO systems with their system age (Y)

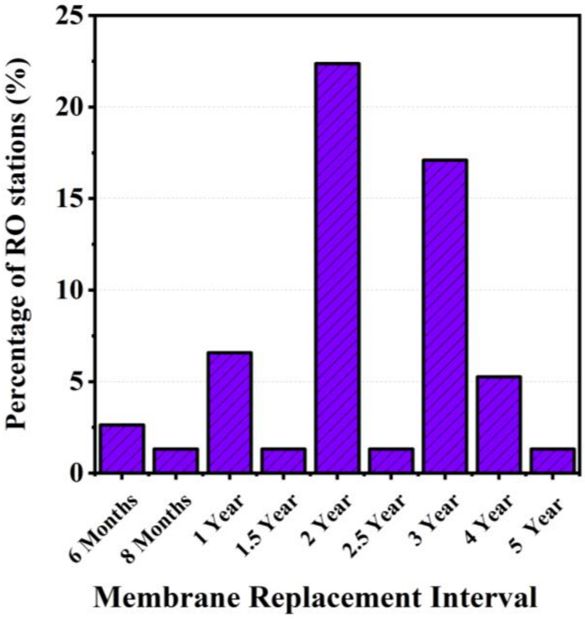

Figure S5. Percentage of the number of CBO operated RO facilities (y-Axis) in NCP with their Membrane Replacement Intervals (x-Axis)

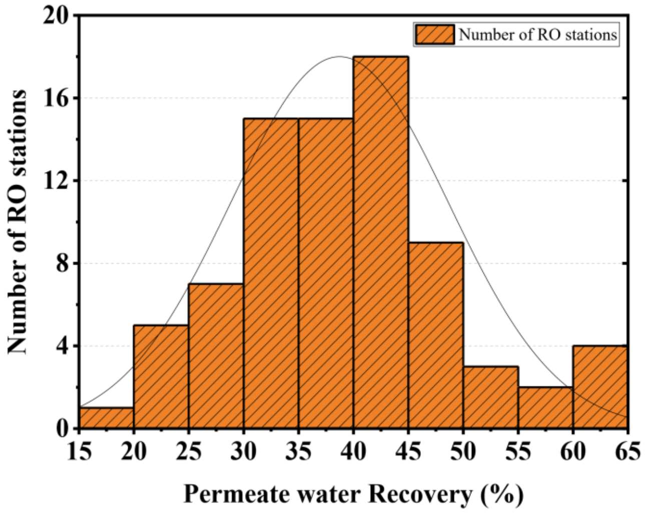

Figure S6. Number of RO plants (y-axis) with the categorization by their Water Recovery as a Percentage (x-axis)

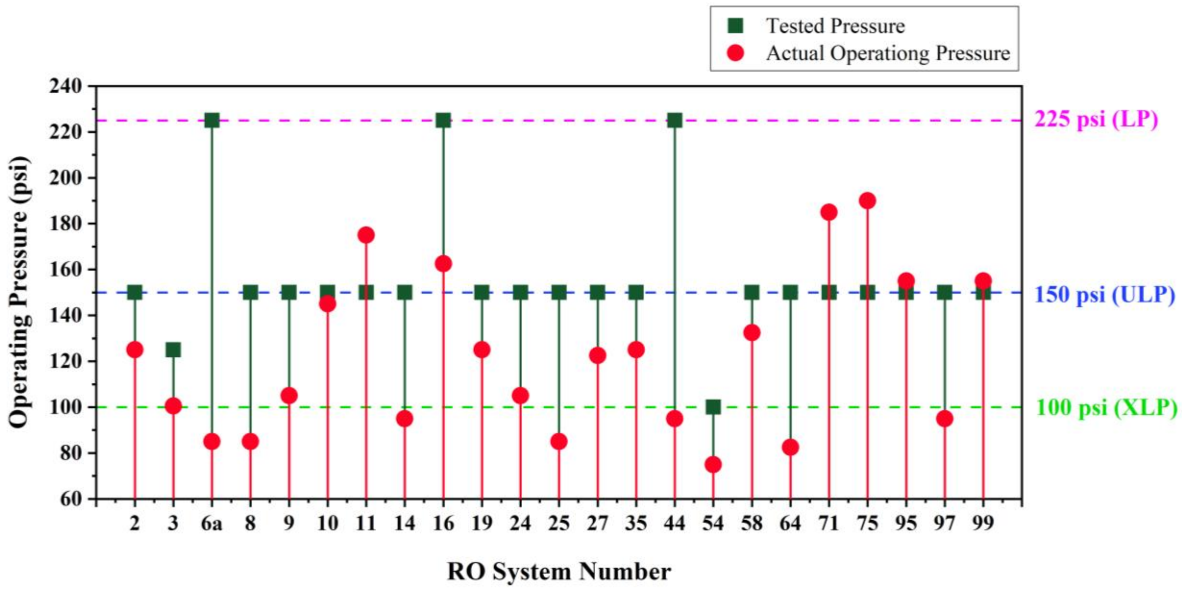

Figure S 7. Tested (by manufacturer) operating pressure vs. actual Operating Pressure of selected RO systems in the NCP

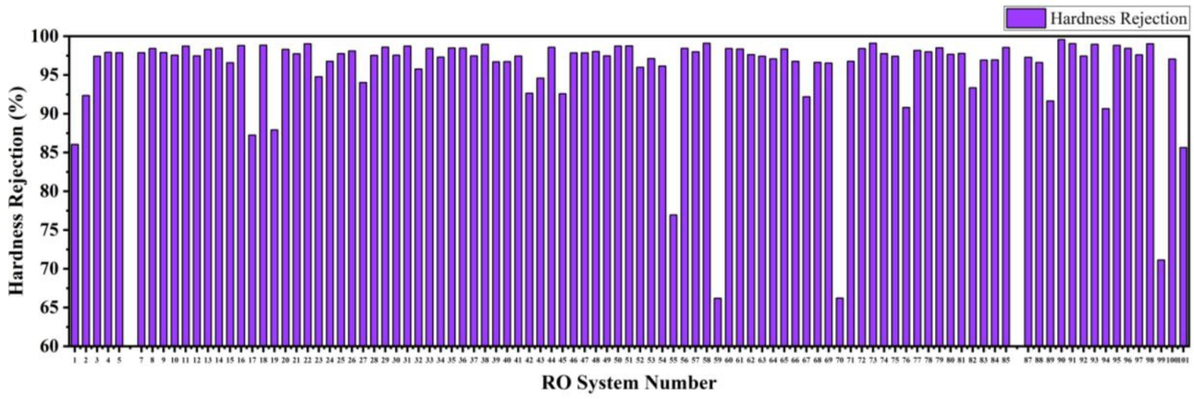

Figure S8. Hardness rejection of each RO station

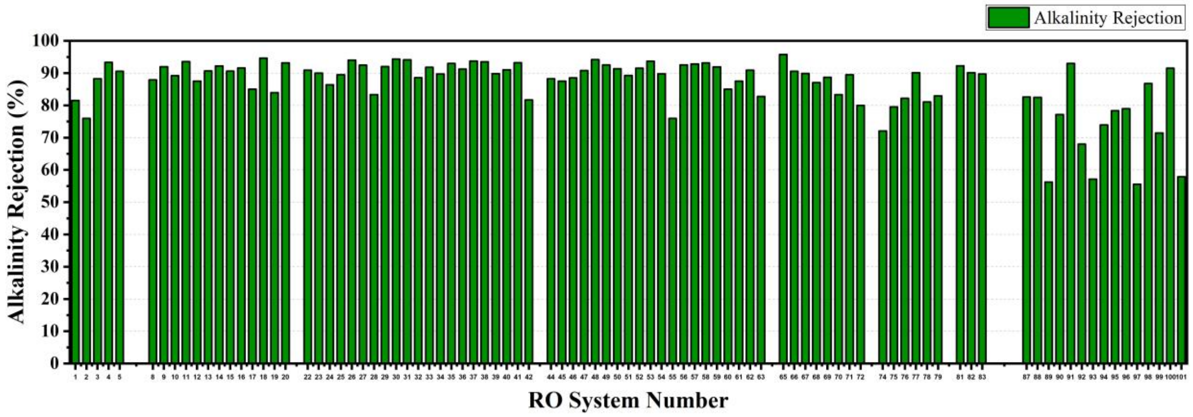

Figure S9. Alkalinity rejection rates of each RO station

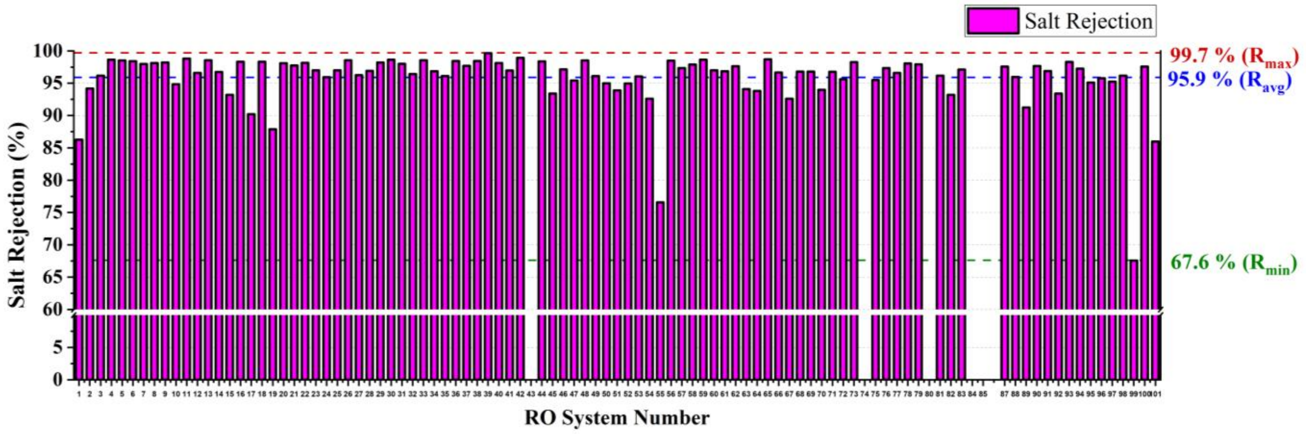

Figure S10. Overall salt rejection for each RO plant (95 ROs) operated under CBOs (x-axis: RO plant number, y-Axis: % salt rejection)

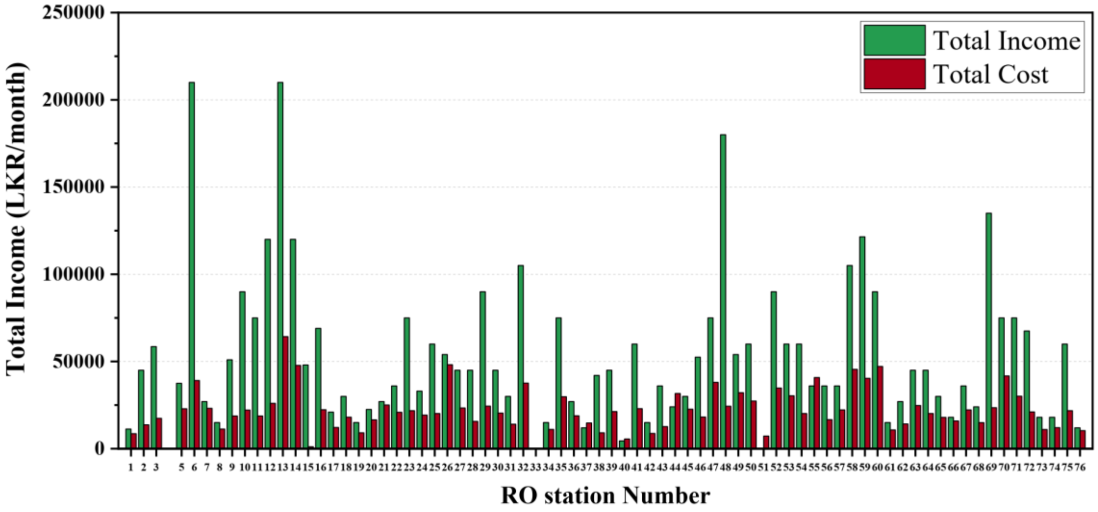

Figure S11. Total income and total operating cost of each RO station in the NCP

Table S 1. RO Product Water Prices with their Operating Companies

RO operated Organizations	RO Water Price LKR / L
CBOs	1 – 1.50
Sri Lankan Navy	Free of charge
Civil Security Department	0.25 – 1
Private and Commercial Organizations/Companies	1.50 – 3.5

Table S2. Diversity of RO system configurations in CBOs in the NCP

No of Stages	RO skids (Pressure vessels)	No of RO elements	RO skids Arrangements	No of RO stations	Illustrations in Figure S2
Single stage	1	1	Single	22	(a)
	2	2	Parallel	1	(c)
2-stage	2	2	Serial	43	(b)
		4		7	(f)
	3	3	Christmas tree	13	(d)
3-stage	3	3	Serial	13	(e)
6-stage	6	6	Serial	1	

Table S3.Specification data of each RO membrane type used in RO stations in the NCP

RO membrane Type/Model	Manufacturer	Salt Rejection	Tested Pressure (psi)	Salt Concentration (mg/L)	Salt Type	Recovery (Per membrane unit) %
ESPA2-LD-4040	Hydranautics	99.6% (99.4% Min)	150	1500	NaCl	15
ESPA 1-LD-4040	Hydranautics	99.4% (99.2% Min)	150	1500	NaCl	15
CPA2-4040	Hydranautics	99.5% (99.2% Min)	225	1500	NaCl	15
LP 21-4040	VONTRON	99.5% (99.3% Min)	225	2000	NaCl	15
ULP21-4040	VONTRON	99.0% (98.5% Min)	150	1500	NaCl	15
ULP 3012	VONTRON	97%	100	500	NaCl	15
RE4040-BN	CSM, South Korea	99.7% (Stabilized)	225	2000	NaCl	15
XLE-4040	DOW Filmtec	99% (Stabilized)	100	2000	NaCl	15
BW30-4040	DOW Filmtec	99.5% (Stabilized)	225	2000	NaCl	15
LC-LE-4040	DOW Filmtec	99.2% (99.1% Min)	125	2000	NaCl	15

Table S4. Influence of feed water chemistry for Ion rejection of ESPA2 membranes

Parameter	Raw Water EC	[Ca]	[Mg]	[Na]	[K]	[Li]	[As]	[Si]
Salt Rejection (%)	0.182	-0.173	0.147	0.243	0.21	-0.078	0.075	-0.086
Ca Rejection %	0.195	0.354	0.189	0.134	0.044	0.068	0.328	-0.058
Mg Rejection %	0.008	-0.261	-0.042	0.093	0.149	-0.244	0.17	-0.275
Na Rejection %	0.264	-0.175	0.131	0.422	0.346	0.121	0.011	0.099
K Rejection %	0.41	0.093	0.465*	0.374	0.363	0.107	0.315	0.004
Li Rejection %	-0.058	0.096	0.124	-0.185	0.013	-0.600**	0.407	-0.155
As Rejection %	-0.158	0.21	0.08	-0.247	-0.376	0.199	0.314	-0.086
Si Rejection %	0.198	-0.07	0.157	0.183	0.249	-0.149	0.259	0.028

**. Correlation is significant at the 0.01 level (2-tailed)
*. Correlation is significant at the 0.05 level (2-tailed)

Table S5. Selected RO membranes for comparison of Individual ion Rejection

Manufacturer	Membrane Type	Number of RO stations
DOW filmtec	BW30-4040	4
Hydranautics	ESPA2-LD-4040	23
VONTRON	ULP21-4040	6

Table S6. Estimation of Production cost for drinking water in a typical RO station in the NCP

Category	Units	Price per Unit / LKR	Number of units	Cost per month (LKR)
Membrane price	1 unit	40000	2	3333
Replacement interval	Year		2	
Antiscalents	1 L	1500		500
Interval	Months per unit		3	
Resin	1 kg	500	50	1042
AC	1 kg	500	30	625
Sand	1 kg	40	25	42
Replacement interval	Year		2	
Sediment cartridge	1 unit	450	1	450
Replacement interval	Months		1	

Pumps repair cost	1 year	5000		417
Operational hours	hour/day		20	
Electricity charge	LKR/month	20000		20000
Capacity	L/day	10000	30	
Operators’ salary	1 month	7500	2	15000
Extra Expenses	1 month	2000		2000
Total monthly cost	LKR/Month			43408
Production cost	LKR/L			**0.145**
